# Cross-Predicting Essential Genes between Two Model Eukaryotic Species Using Machine Learning

**DOI:** 10.3390/ijms22105056

**Published:** 2021-05-11

**Authors:** Tulio L. Campos, Pasi K. Korhonen, Neil D. Young

**Affiliations:** 1 Department of Veterinary Biosciences, Melbourne Veterinary School, Faculty of Veterinary and Agricultural Sciences, The University of Melbourne, Parkville, VIC 3010, Australia; tulio.campos@fiocruz.br (T.L.C.); pasi.korhonen@unimelb.edu.au (P.K.K.); 2 Bioinformatics Core Facility, Instituto Aggeu Magalhães, Fundação Oswaldo Cruz (IAM-Fiocruz), Recife 50740-465, PE, Brazil

**Keywords:** essential genes, eukaryotes, *Drosophila*, *Caenorhabditis*, machine learning, predictions

## Abstract

Experimental studies of *Caenorhabditis elegans* and *Drosophila melanogaster* have contributed substantially to our understanding of molecular and cellular processes in metazoans at large. Since the publication of their genomes, functional genomic investigations have identified genes that are essential or non-essential for survival in each species. Recently, a range of features linked to gene essentiality have been inferred using a machine learning (ML)-based approach, allowing essentiality predictions within a species. Nevertheless, predictions between species are still elusive. Here, we undertake a comprehensive study using ML to discover and validate features of essential genes common to both *C. elegans* and *D. melanogaster*. We demonstrate that the cross-species prediction of gene essentiality is possible using a subset of features linked to nucleotide/protein sequences, protein orthology and subcellular localisation, single-cell RNA-seq, and histone methylation markers. Complementary analyses showed that essential genes are enriched for transcription and translation functions and are preferentially located away from heterochromatin regions of *C. elegans* and *D. melanogaster* chromosomes. The present work should enable the cross-prediction of essential genes between model and non-model metazoans.

## 1. Introduction

The elegant worm, *C. elegans* (*CE*), and the fruit fly, *D. melanogaster* (*DM*), belong to the Superphylum Ecdysozoa, comprising animals that moult [1]. This large taxonomic group includes a range of parasitic worms and arthropods, some of which are causative agents or vectors of infectious diseases [2,3]. Taken together, such parasites and vectors inflict substantial socioeconomic damage worldwide by causing or transmitting disease and/or affecting animals or crops [4,5]. *CE* and *DM* are used as models to study many fundamental biological and molecular aspects of multicellular organisms because they have short life cycles, are readily maintained in laboratory, reproduce in large numbers, and can be genetically altered using established knockout and knockdown approaches [6]. The availability of their genomes and experimental methods for both species have facilitated large-scale studies of molecular function and identified key genes that underpin survival (i.e., essential genes) [7,8]. The genome-wide characterisation of essential genes in a broad range of eukaryotic species is paramount to understanding the fundamental machinery that governs life [9,10,11]. This understanding could provide a foundation for innovative biological and biotechnological applications, such as the development of novel and precise intervention strategies against socioeconomically important parasites and/or disease vectors. However, functional genomics tools for such non-model organisms are usually not established [12], and large-scale discovery of essential genes laborious, time-consuming, and costly [13]. An alternative approach would be to utilise high-genomic and transcriptomic data sets available for model species (e.g., [14,15]) to identify key features that define gene essentiality between or among eukaryotic species.

Despite the technical advances and the wealth of ‘omics data available for *CE* and *DM*, the features defining gene essentiality are poorly understood. Computational methods, such as machine learning (ML), are now being used to explore such features for effective computational prediction [16]. Previous studies have attempted to predict essential genes within eukaryotic species by exploring features based on homology, protein–protein interaction (PPI) network analysis, and/or sequence characteristics [13,16,17,18,19,20]. These studies have shown that better predictions by ML approaches can be achieved by combining predictive features from multiple data sources. For example, a recent study combined features derived from gene sequence, ontology, and PPI networks to improve the performance of essential gene predictions for *DM* [21]. In recent publications by our research group, we harnessed the abundance of ‘omics data sets publicly available for *CE* and *DM* to discover strong predictors for essential genes, and showed that ML-based predictions were accurate within each species [22,23]. Despite this, reliable predictions between or among eukaryotic species remains challenging. In a first attempt, we showed that protein sequence-derived features were useful for cross-species predictions of essential genes [20]. Nevertheless, there is no comprehensive study exploring the wealth of features now available for *CE* and *DM* to discover consensus gene essentiality predictors for the accurate prediction of essential genes between or among species. Such a study would enhance our understanding of essential genes in non-model ecdysozoans, including socioeconomically important parasites [24]. In the present study, we harnessed extensive feature sets recently defined for *CE* and *DM* [22,23] to undertake a comprehensive investigation of common features and to predict essential genes *between* these two model species using an ML-based approach.

## 2. Results

### 2.1. A Selection of Strong Predictive Features of Essential Genes for CE and DM Identified by Predictions within Species

We obtained 55,694 features for 18,461 *CE* genes, and 33,759 features for 11,580 *DM* genes compiled by Campos et al. [22,23]. Of these features, 1391 were present in both *CE* and *DM* feature sets, of which 8 were readily standardised. Another eight features were added to those, including two calculated from ‘expressed sequence tag’ (EST) data mapped to genomes, two histone modifications markers defined by ChIP-seq data (H3K4me3 and H3K27me3—trimethylation of the H3 protein at lysin 4 or 27, respectively), and 4 PPI network centrality features (see Section 4). Therefore, we compiled a total of 1399 features for each *CE* or *DM* gene that were used for downstream analyses.

For *CE* and *DM* data sets, we selected 621 *CE*/359 *DM* genes with the highest (>0.7) and 16,690 *CE*/9579 *DM* genes with the lowest (<0.1) probabilities of being essential, defined by previous ML approaches [22,23]. Using this selection of genes and their 1399 corresponding features, we employed a strategy for essential gene predictions and cross-validation within species using random subsampling of the data (from 10 to 90% of the data for training, with remaining data used for testing, with 10% increments), followed by feature selection (consensus between elasticNet and ensemble sparse partial least square [SPLS] methods), training and evaluation of six ML algorithms, and a background model for each subsample (see Section 4; [23]).

**Figure 1:**
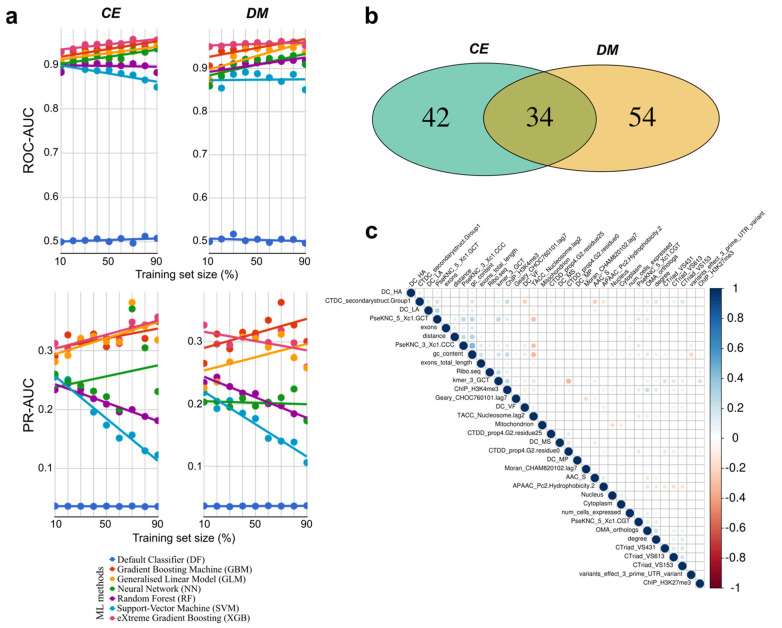
Evaluation of essential gene predictions within species using standardised/consensus features for *Caenorhabditis elegans* (*CE*) and *Drosophila melanogaster* (*DM*). (**a**) For each species, subsets (10–90%, 10% increments) of high-confidence essential and non-essential genes [22,23] were randomly selected (*x*-axis) to train six machine learning approaches/methods, following a feature selection strategy. Prediction performance (ROC-AUC and PR-AUC) was evaluated using the remaining data. (**b**) Venn diagram of the best-predictive features identified for *CE* and/or *DM*. (**c**) Pairwise correlations between the 34 best-predictive features identified for both species.

By evaluating the systematic ML approach within species using selected features for *CE*, the calculated receiver operating characteristic (ROC)—area under the curve (AUC) for the ML models were consistently >0.9 for the gradient boosting machine (GBM), eXtreme gradient boosting machine (XGB), generalised linear model (GLM), and neural network models (NN). The performances increased steadily as more data were included in the training sets (Figure 1a). For random forest (RF) models, the ROC-AUC performance was stable around ~0.9. For support vector machine (SVM), ROC-AUC decreased from ~0.9 to ~0.85 (Figure 1a). Regarding precision-recall (PR)-AUC, the performance increased from ~0.3 to ~0.35 for GLM, GBM, and XGB; decreased for RF (~0.24 to ~0.18) and SVM (~0.26 to ~1.2); and was highly variable for NN, ranging between ~0.2 and ~0.35 (Figure 1a). Overall, the XGB and GBM methods were the best performers. We confirmed that those performances were robust even when using a non-redundant set, whereby genes with similar sequences (>25% identity) at the protein level were excluded from the analysis (Supplementary Material Figure S1). 

In total, 76 features were selected as best predictors for *CE* (Supplementary Material Table S1; Figure 1b). Among those, num_cells_expressed (number of cells where the gene is expressed, obtained from single-cell data), OMA_orthologs (number of genes present in the ortholog group, obtained from the Orthologous Matrix database), ChIP_H3K4me3 (histone marker overlapping a gene region, obtained from ChIP-seq data), and ‘exons’ (number of exons for a gene) were among the highest (top 10) feature importances for predictions in all six ML models. Other selected features among the 76 were derived from DNA or protein sequences, such as GC content, AAC_S (ratio of serine residues in a protein), predictions of protein subcellular localisations (particularly cytoplasm and nucleus), and presence of a signal peptide. Additional predictive features identified were derived from single-nucleotide polymorphism (SNP) analysis (e.g., number of SNPs and variant effect on the 3’ UTR) and two PPI network features (‘degree’ and ‘betweenness’ centrality). 

For *DM*, the ROC-AUC curves achieved for the systematic ML predictions within species for most models were also >0.9, increasing in performance as more data were added to the training sets, except for SVM, which ranged between ~0.85 and ~0.9 (Figure 1a). Regarding the PR-AUC, the XGB and GBM models achieved between ~0.26 and ~0.35, GLM between ~0.22 and ~0.31, NN was stable around ~0.2, while for RF and SVM it decreased from ~0.24 to ~1.8 and from ~0.22 to ~0.11, respectively (Figure 1a). Overall, XGB and GBM were also the best performing models based on the ROC-AUC and PR-AUC metrics. Again, the observed performances were robust using a non-redundant set (Supplementary Material Figure S1). For *DM*, 88 best features were selected as best predictors (Supplementary Material Table S2; Figure 1b). Among the best predictors, ‘exons’, num_cells_expressed, OMA_orthologs, EST_BLAST (expressed sequence tag data mapped to the genome), ChIP_H3K4me3, subcellular localisations (membrane, nucleus, cytoplasm), GC content, and ‘degree’ of centrality ranked highest.

**Figure 2:**
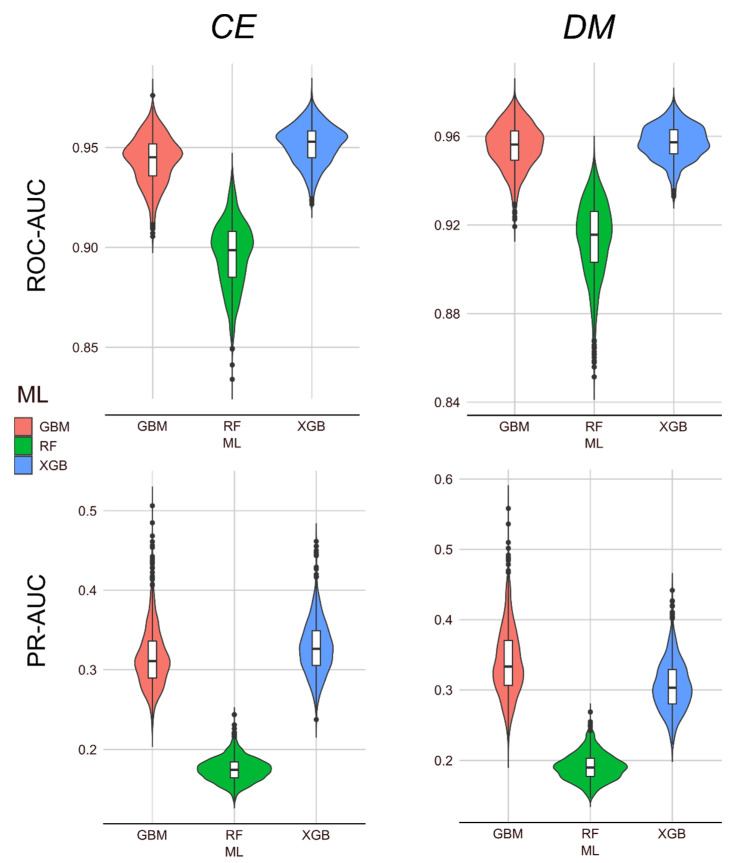
Bootstrap ML approach within species. Violin plots depicting the ROC-AUC and PR-AUC values (*y*-axis) for bootstrap approaches for *CE* (**left**) and *DM* (**right**) using the 34 best-predictive features identified for these species. A total of 1000 random selections containing 90% of the genes (essential and non-essential) were used to train the gradient boosting machine (GBM), random forest (RF), and eXtreme gradient boosting (XGB) models (*x*-axis), using the remaining 10% of the genes for testing.

In total, 34 features were identified as best predictors for both *CE* and *DM* analyses (Figure 1b,c): 21 were derived from DNA or protein composition/autocorrelation, 3 from genomic data (‘exons’, ‘exons_total_length’, and ‘distance’ from the chromosome centre), 3 from subcellular localisation (cytoplasm, nucleus, mitochondrion), 2 from ChIP-seq data (ChIP_H3K4me3 and ChIP_H3K27me3), one from variant effect at the 3’-prime UTR (variants_effect_3_prime_UTR_variant) based on SNP data analysis, 1 from Ribo-seq data mapped to the genome (Ribo.seq), as well as 3 others derived from other data sets (num_cells_expressed, OMA_orthologs and ‘degree’ of centrality). The pairwise correlations between these 34 features were usually low (<0.2), and occasionally moderate (ranging from 0.2 to 0.45) (Figure 1c). We further assessed the performance of the XGB and GBM models (RF included as control) using a bootstrap approach within species, where we randomly selected 90% of the data for training and 10% for testing, 1000 times. For *CE*, the ROC-AUC ranged mostly between ~0.90 and ~0.97 with a median of ~0.95, whereas RF was between ~0.85 and ~0.95 with a median ~0.90. The PR-AUC ranged mostly from ~0.25 to ~0.4 with a median ~0.33, whereas RF ranged between ~0.1 and ~0.22 (Figure 2). Compared to *CE,* a markedly similar performance (ROC-AUC and PR-AUC) was observed for *DM* (Figure 2).

### 2.2. Select Features and ML Models Enable Essential Gene Predictions between CE and DM

**Figure 3:**
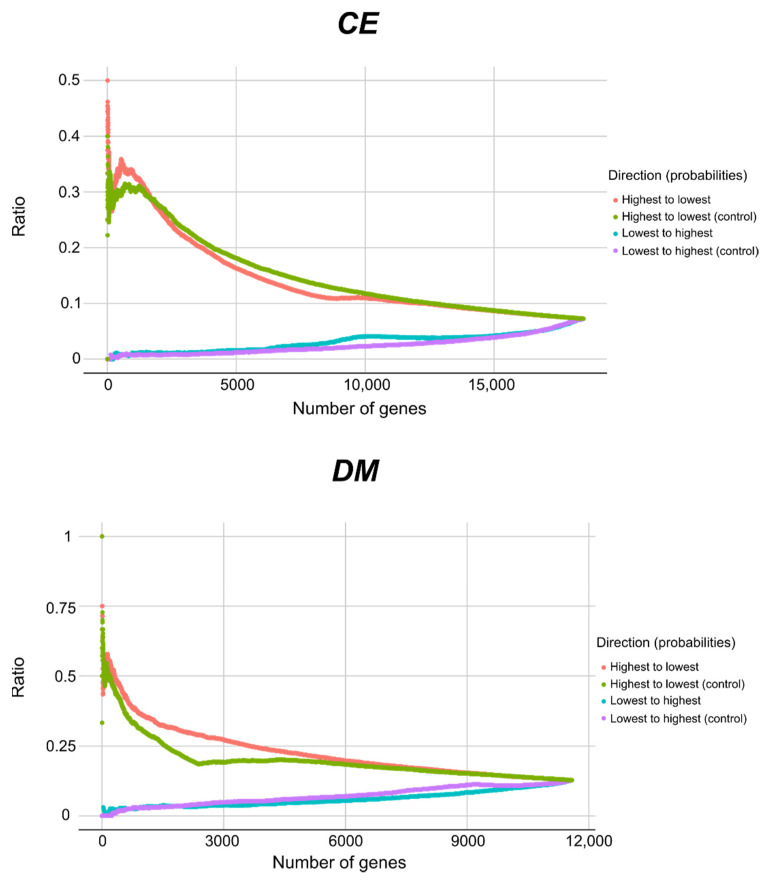
Validation of cross-species essential gene predictions. Following the predictions between species using extreme gradient boosting (XGB) models trained with select features identified in the within-species approach, genes were ranked by the essentiality probabilities. The ranked genes (*x*-axis) were cumulatively searched from the highest to the lowest probabilities, as well as from the lowest to the highest against the GExplore database [25] (*DM* data used to predict *CE*—**top**) and GenomeRNAi database [26] (*CE* data used to predict *DM*—**bottom**). Cumulative ratios of genes with a ‘lethal’ phenotype reported in each database were calculated (*y*-axis). Ranked genes following training and prediction within species were used as controls.

We selected the best predictive features that were identified for both *CE* and *DM* to train XGB models and to perform predictions between species. Then, the XGB model trained with *DM* data was used to predict essential genes in *CE*. The essentiality probabilities varied from zero to ~0.63. These probabilities showed a Spearman correlation of ~0.55 with rankings defined by previous predictions in *CE* [22]. The essentiality probabilities for the ranked genes decreased rapidly from ~0.63 to ~0.1 after the first 1000 genes and approached zero after the first 3000 genes (Supplementary Material Table S3). For validation, the ranked genes were cumulatively searched against independent functional data for *CE* (GExplore [25]). The ratio of genes with a lethal phenotype decreased from ~0.5 to ~0.07 when searching genes from the highest to the lowest probabilities and increased from 0 to ~0.07 when searching from the lowest to the highest (Figure 3—top). The observed pattern of ratios was similar to the control experiment ratios, in which the same analysis was employed using the XGB model trained with *CE* data. 

Subsequently, the XGB model, trained with *CE* data, was used to predict essential genes in *DM*. The essentiality probabilities for all genes varied from 0 to ~0.68. These probabilities showed a Spearman correlation of ~0.40 with ranked genes defined by previous predictions in *DM* [23]. Then, all *DM* genes were ranked by the probability for essentiality defined by the models. These probabilities decreased rapidly from ~0.68 to ~0.1 after 1000 genes ranked and ordered by essentiality probability, approaching zero after ~3000 genes (Supplementary Material Table S4). As a validation step, the ranked genes were cumulatively searched against independent functional data (GenomeRNAi [26]) for *DM* (Figure 3—bottom). The ratio of genes with a lethal phenotype decreased from 1 to ~0.13 when cumulatively searching for genes from the highest to the lowest probabilities and increased from 0 to ~0.13 when searching from the lowest to the highest. The pattern of ratios was similar to the control experiment, in which the same analysis was employed using the XGB model trained with *DM* data.

### 2.3. Visual Representation of Cross-Species Gene Essentiality Probabilities along the CE and DM Genomes

**Figure 4:**
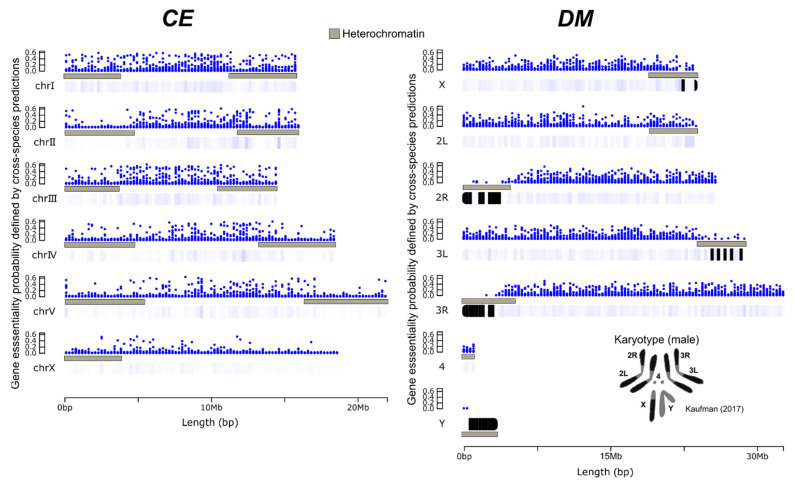
Distribution landscape of essentiality probabilities along *CE* and *DM* chromosomes. Essentiality probabilities defined by the XGB models (*x*-axis) for the cross-species predictions were plotted for each gene coordinate (*y*-axis). Heterochromatin regions defined by previous studies for *CE* [27] and *DM* [28] are depicted in grey. Heatmaps below each chromosome show the areas with higher (dark blue) and lower gene densities (white), and no data (black). Additional information on karyotype for *DM* [28] shows the relationship between chromosome segments.

We assessed the landscape of essential gene probabilities by plotting the essentiality probabilities from the cross-species predictions along the chromosomal DNA sequences of *CE* or *DM* genomes. For *CE*, genes with higher probabilities of being essential (>0.5) appeared to be preferentially located in chromosomes “I” and “III”, followed by “II”, “IV”, and “V” (Figure 4). Moreover, the sex chromosome “X” seemed to contain fewer genes with a high probability of being essential, compared to other chromosomes. Overall, genes with higher probabilities of being essential were found in or near the centre of chromosomes, away from the regions experimentally defined as heterochromatin in previous studies [27,28]. For *DM*, genes with higher probabilities also appeared to be preferentially located away from heterochromatin regions of autosomal chromosomes (particularly “2R” and “3R” segments) and telomeric regions (edges) of “X” (Figure 4). Excluding the heterochromatin regions, local hotspots of genes with the highest essentiality probabilities were relatively evenly distributed along chromosome segments “2L”, “2R”, “3L”, “3R”, and “X”. Moreover, genes with higher probabilities were less likely to be found on chromosomes “4” and “Y”.

### 2.4. Gene Ontology (GO) Enrichment and Functional Clustering Analyses Confirm Important Roles of Essential Genes

We conducted GO and functional clustering analyses using selections of genes with both highest and lowest probabilities of essentiality established by the cross-species predictions. For 500 *CE* genes with the highest probabilities, the most significantly enriched clusters were associated with ribosome/translation (24–91 genes), nucleotide/ATP-binding (88–119 genes), cell division/mitosis (8–32 genes), aminoacyl-tRNA synthetase/ligase (9–33 genes), and cytoskeleton (12–32 genes) (Supplementary Material Table S5). Of those 500 genes, 193 (38.6%) are single-copy genes and 37 (7.4%) did not have an ortholog in *DM* according to the Ensembl database [29]. Similarly, the 500 most likely essential genes in *DM* were enriched for: ribosome/translation (15–59 genes), mRNA splicing/spliceosome (25–41), transcription/regulation (32–49 genes), nucleotide/ATP-binding (24–60 genes), and mRNA splicing regulation (7–13 genes) (Supplementary Material Table S6). Of those 500 genes, 247 (49.4%) are single-copy genes and 82 (16.4%) did not have an ortholog in *CE*.

For the 500 most likely non-essential genes for *CE*, enriched functions were: MATH/TRAF proteins (30–31 genes), F-box domain (13–36 genes), transmembrane/membrane (133–135 genes), dsRNA transport/SID1 transmembrane (4 genes), and BTB/protein homo-oligomerisation (6–14 genes) (Supplementary Material Table S5). Of those, 119 (23.8%) were single-copy genes and 112 (22.4%) did not have an ortholog in *DM*, according to the Ensembl database. For *DM*, enriched functions for the 500 most likely non-essential genes were: transmembrane/membrane (178–197 genes), olfactory/odorant (9–47 genes), peptidase S1/proteolysis (10–38 genes), transmembrane/substrate transporter (7–25 genes), and lipase/ester hydrolase activity (9–19 genes) (Supplementary Material Table S6). Of those, 204 (40.8%) were single-copy genes and 183 (36.6%) did not have an ortholog in *CE*.

## 3. Discussion

Here, we comprehensively demonstrate that the cross-prediction of essential genes between two well-characterised model organisms is possible using an ML-based approach. This was achieved by discovering strong consensus predictors of essential genes in each species. The present work provides prospects for the prediction and validation of essential genes in non-model ecdysozoan species. 

The discovery/confirmation of consensus predictors of essential genes within *CE* and *DM* was instrumental for the successful cross-species predictions using ML. To enable essentiality predictions between these species, 1399 features were assessed [22,23], and a selection of 34 was found to be the most predictive in both species. Among those, features derived from scRNA-seq (early developmental stage [30,31]) and ChIP-seq (H3K4me3—associated with promoter regions [32]; H3K27me3—associated with transcriptional repression [33]) were confirmed to be strong essential gene predictors. Moreover, select genomic and sequence-derived features, such as protein size, number of exons, subcellular localisation, and a selection of DNA and protein sequence features, were highly predictive. We confirmed that ‘degree’ of centrality in PPI networks, and amino acid sequence conservation (orthologs [34]) were also important predictors, as suggested by other studies in multiple species [13,19,21,35,36]. Collectively, these findings indicate that essential genes have specific genomic (sequence/location), transcriptomic/proteomic (expression), epigenetic (regulatory), network (interactions), and conservation (orthologs) signatures that can be harnessed for predictions both within and between species using ML. Therefore, these critical aspects of gene essentiality and their causal relationships should be explored in the future. 

Consistent and accurate prediction performance was achieved by the ML models trained with the strongest consensus predictors of essential genes identified in *CE* and *DM*. Overall, the systematic ML approaches employed within species with the best predictive features for the individual species showed that the prediction performances based on ROC-AUC were high (>0.9), and the PR-AUC was ~0.3 (Figure 1a). The results for the ROC-AUC were consistent with previous analyses [22,23] whereas a loss in PR-AUC performance was observed. Nevertheless, the overall performance was robust even when using only the 34 best-predictive features common to both species, as demonstrated by the bootstrap approach (Figure 2). Moreover, the low pairwise correlations observed among the 34 best-predictive features (Figure 1c) suggest that they are both non-redundant and complementary for essentiality predictions using ML. In terms of ML performance, the boosting methods (XGB and GBM) were the best based on threshold-independent metrics (ROC-AUC and PR-AUC), confirming the high accuracy and robustness for essentiality predictions using such approaches. Boosting methods usually outperform deep learning methods in the context of well-structured and tabulated data; see [37]. However, there is still potential to improve the PR-AUC. The PR-AUC metric considers only the prediction of essential genes (positives), but it is informative for imbalanced data sets [38]. As the total number of essential genes encoded in a genome is far fewer than that of non-essential genes in both species, there is a higher chance of predicting false positives, thereby affecting the PR-AUC results. Indeed, imbalanced data sets are common in ML approaches, potentially affecting the values and interpretation of different performance metrics [38]. Oversampling (e.g., Synthetic Minority Oversampling TEchnique—SMOTE) or undersampling techniques have been proposed as solutions [39,40], and we have carefully considered them here. However, there is no consensus that such approaches resolve the problem, as they may introduce unnecessary bias [39,40]. Therefore, we opted to use imbalanced data sets in our systematic training and test evaluations as they reflect the reality of the biological problem (i.e., gene essentiality). Indeed, class imbalance is expected in both the data available for ML training and new sets of genes to be predicted, particularly in the context of gene essentiality. Hence, important information from the majority class (non-essential) was not removed unnecessarily by under-sampling, or bias towards the majority class was not introduced by adding artificial samples through oversampling. Previously, we showed that high prediction performance was achievable within species, despite using imbalanced data sets [22,23]. Therefore, it is possible that there are features of essential genes that are species or taxon specific. For example, Campos et al. [20] showed that essential genes of distantly related species can have quite distinct protein sequence features. Nonetheless, species-specific features would be challenging to use for cross-species predictions.

Following the essential gene predictions between species using XGB, the genes were ranked by their predicted probabilities for essentiality. Interestingly, the genes most likely to be essential exceed the probability of 0.7. This may also be an effect of the imbalanced data sets and/or a result of the evolutionary distance between *CE* and *DM* [1]. Nonetheless, we ranked the genes by probability of essentiality to validate the predictions using independent functional data sets for each species (Figure 3) and these analyses clearly showed that the probabilities correlate with lethal phenotypes. Our findings were consistent with previous results [22,23], demonstrating that the large-scale predictions between species are possible using this ranking approach. 

The essentiality probabilities per gene defined by predictions between species and plotted on chromosomes showed that there were preferential genomic locations for essential genes. However, the locations were markedly different between *CE* and *DM*. For *CE*, the most likely essential genes tend to be located in or near the centre of autosomal chromosomes. For *DM* “hotspots” for essential genes are more widely distributed, including on the sex chromosome “X”. These differences in the distribution of essential genes may also be linked to their distinct karyotypes and estimated heterochromatin/euchromatin regions [27,28]. In addition, the chromatin and centromere/holocentromere organisations are markedly different between *CE* and *DM* [41,42]. These aspects affect DNA packaging and transcriptional regulation and remain to be deeply explored in the context of gene essentiality. In addition, the roles of essential genes in sex determination and reproduction remain to be investigated in these species, particularly considering the very distinct reproductive modes for *CE* (usually selfing) and *DM* (outcrossing) [43].

From a functional perspective, we found that the most enriched functions in clusters of the 500 most likely essential genes in either species were primarily associated with ATP-binding, RNA splicing/processing, and translation. These results provide more evidence that most essential genes carry out fundamental intracellular activities, in accordance with their preferred subcellular localisations (nucleus/cytoplasm/mitochondrion). Surprisingly, only 38.6% (*CE*) and 49.4% (*DM*) of the essential genes were found to be single copy, challenging the assumption that paralogous genes are redundant in function [44]. For example, previous functional studies in *CE* and mouse have shown that a large number of duplicated genes are essential for these species [44,45]. On the other hand, a large proportion of the single-copy genes, non-essential genes identified here, did not have an ortholog in the alternative species. Therefore, these findings suggest that the identification of orthologs between distantly related species appears to be more important than quantifying paralogs within species for the purpose of essential gene predictions or prioritisations.

In conclusion, the present work has demonstrated, for the first time, that the accurate large-scale cross-species prediction of essential genes *between* a worm and a fly is possible employing a well-defined set of informative features. These findings and insights provide a foundation for the ML-based prediction of gene essentiality in non-model organisms, such as parasites and vectors of infectious diseases, with possible biotechnological implications and applications in the future.

## 4. Materials and Methods

### 4.1. Defining Feature Sets

Genes ranked by essentiality probabilities, and their corresponding features derived from genomic, transcriptomic, and proteomic information, for *CE* and *DM*, were obtained from previous publications [22,23]. Features not present in the data sets of both species, and the features that could not be standardised between the species, were filtered out. 

Novel features derived from EST, histone modification markers, and PPI were added to the feature set. EST sequences data was obtained for *CE* [14] and *DM* [46] and combined into a single FASTA file. Using this FASTA file, two features were generated by aligning the EST data to *CE* and *DM* gene sequences: the first by counting the number of significant hits per gene using BLAST v.2.10.1+ (parameters: -evalue 1e^−10^ -ungapped) and the second by counting significant hits using BLAT v.35x1 (default parameters) [47]. We also obtained features from ChIP-seq data obtained from modENCODE [48] for *CE* and *DM* (histone modifications H3K4me3 and H3K27me3; data sets 3811, 4987, 5163, 5166). Sequencing quality was checked using FastQC (https://www.bioinformatics.babraham.ac.uk/projects/fastqc, accessed on 18 September 2020), adapter-trimmed reads of individual chromatin-immunoprecipitated and input samples were aligned using BWA v.0.7.10-r789 [49] with default parameters, and peaks were called using MACS2 [50], parameters: -f BAM -g [ce/dm] -B -q 0.01. The number of peaks overlapping each *CE* or *DM* gene coordinates (GFF; obtained from WormBase [14] and FlyBase [15]) was calculated using BEDTools v.2.26 [51] *intersect*. Next, for each *CE* or *DM* gene (by Ensembl identifier), we quantified the number of orthologs present in their orthologs groups found in the “OMA groups” and “Mapping to Ensembl” files from the Orthologous Matrix Database [34], and added this information as a feature. Finally, we used the STRINGdb (http://doi.org/10.18129/B9.bioc.STRINGdb, accessed on 21 October 2020) and igraph (http://igraph.org, accessed on 21 October 2020) packages for R v.3.6 (versions for all of the libraries used here are available in a git repository —see Data Availability Statement) to obtain PPI data and features (‘degree’, ‘betweenness’, and ‘closeness’ centrality), respectively. 

### 4.2. Feature Selection, ML Training, and Evaluation within Species Using Standardised Data

We established the most probable essential genes (probability > 0.7) and non-essential genes (probability < 0.1) for *CE* and *DM*. Then, we used these genes and their corresponding features to train and evaluate six ML approaches (GBM, GLM, NN, RF, SVM, XGB) with hyperparameter optimisation using the “caret” package for R v.3.6 (https://topepo.github.io/caret, accessed on 10 March 2021) as defined by Campos et al. [23] but used the new set features common to both *CE* and *DM* [23] included for this study. Briefly, random samples containing 10 to 90% of *CE* data or *DM* data (with 10% increments) were used to perform feature selection and training of six ML models, with the remaining 90 to 10% as test sets. The prediction performances for each sub-selection were evaluated on the test sets using ROC-AUC and PR-AUC metrics and plotted using “ggplot2” for R (https://ggplot2.tidyverse.org, accessed on 10 March 2021). Then, 100% of the *CE* or *DM* feature set was used to train the ML approaches with their respective best-predictive features identified within species. Then, we established the strongest predictors by ranking and evaluating the median feature importance (“caret” package) among the final ML models. This systematic evaluation was also employed using a non-redundant set, whereby, for each species, genes were clustered (>25% protein sequence identity) using USEARCH v.11 (https://www.drive5.com/usearch, accessed on 18 March 2021), and only the centroid sequences were retained and used for feature selection, ML training/testing, and performance evaluation. We identified the best-predictive features within species for *CE* or *DM* based on the feature selection, as well as the best-performing ML approaches based on ROC-AUC and PR-AUC metrics. Finally, we used such features and ML approaches to carry out a 1000-boostrap approach [23] using 90% of the data for training and 10% for testing, evaluating the same metrics.

### 4.3. Employing and Evaluating the ML Approach for Predictions between Species

For the prediction between species, we selected the best-performing ML approach from within species prediction and the best-predictive features separately for *CE* and *DM*. Then, complete feature sets of each species were used to train the model and predict essentiality for all genes of the alternative species. Once predicted, genes were ranked by their essentiality probabilities defined by the ML models. We plotted the essentiality probabilities on *CE* and *DM* chromosomes using ‘chromoMap’ for R v.4.0 (https://lakshay-anand.github.io/chromoMap, accessed on 8 April 2021). The prediction performances of the ML models were evaluated by using Spearman correlations between the essentiality probabilities established elsewhere [22,23] and the novel prediction probabilities established here. As a further validation step, we used the genes ordered by their essentiality probabilities. Using these ranked lists, we cumulatively calculated and plotted the ratios of genes linked to lethal phenotypes based on independent functional genomic data [25,26]. Such a validation approach has been established and successfully used in other studies [22,23]. 

### 4.4. Gene Ontology Analyses with Functional Annotation Clustering

For *CE* or *DM*, we selected 500 genes with the highest essentiality probabilities of each species determined by the XGB model trained with the cross-species models and performed functional enrichment and clustering analysis using the database DAVID [52] v. 6.8 with ‘medium’ stringency, selecting the five most enriched clusters. For each of those lists, we identified single-copy genes using the “BioMart” tool of the Ensembl database [29]. In addition, we performed the same analysis using a selection of 500 genes with the lowest essentiality probabilities.

## Data Availability

The data and code used in the present study as well as information on software versions and R libraries are available at: https://bitbucket.org/tuliocampos/essential_CEDM. A static version of the package containing the code and data linked to this publication is available at: https://10.6084/m9.figshare.14273369.

## Supplementary Materials

The following are available online at https://www.mdpi.com/article/10.3390/ijms22105056/s1, Figure S1: Evaluation of essential gene predictions within species using consensus features and a non-redundant gene set, Tables S1–S6: Supporting information on predictive features and their relative importances, predicted gene essentiality probabilities, and GO analyses.
