# Supplementary material for: Cross-Predicting Essential Genes between Two Model Eukaryotic Species Using Machine Learning
Source: Int J Mol Sci. 2021 May 11;22(10):5056. doi: 10.3390/ijms22105056 (PMC8150380; doi:10.3390/ijms22105056)
Supplement: Supplementary file 1 [file ijms-22-05056-s001.zip › Supplementary_Figure_S1.pdf]

Supplementary Figure:

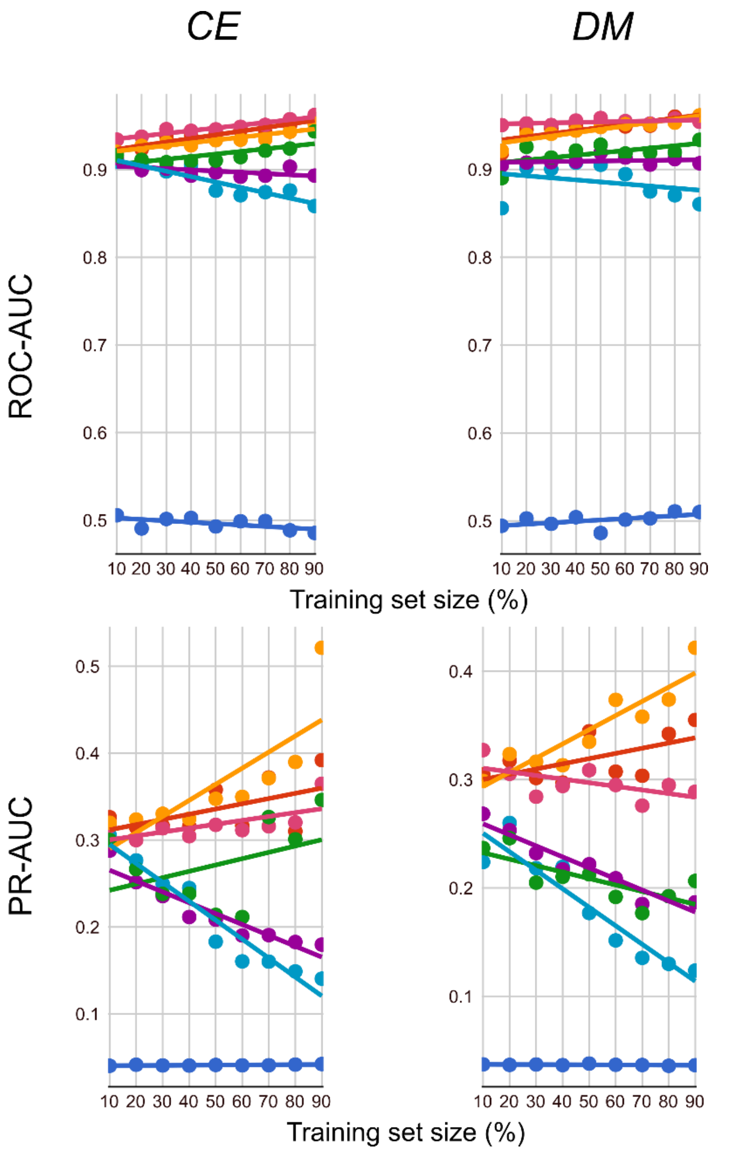

Figure S1. Systematic evaluation of essential gene predictions within species using a non-redundant set. High-confidence essential and non-essential genes [22,23] (Campos et al., 2020a,b) were obtained, genes with protein sequence identity > 25% were clustered, and only the genes representing centroids were retained. Then, for each species, subsets (10-90%, 10% increments) of non-redundant essential and non-essential genes were randomly selected (x-axis) to train of six machine learning approaches/methods, following a feature selection strategy. Prediction performance (ROC-AUC and PR-AUC) was evaluated using the remaining data.
